# Metabolic Syndrome and Functional Fitness Abilities

**DOI:** 10.3390/jcm10245840

**Published:** 2021-12-13

**Authors:** Laura Gallardo-Alfaro, Maria del Mar Bibiloni, Emma Argelich, Escarlata Angullo-Martinez, Cristina Bouzas, Josep A. Tur

**Affiliations:** 1 Research Group on Community Nutrition & Oxidative Stress, Guillem Colom Building, Campus, University of Balearic Islands-IUNICS, IDISBA & CIBEROBN (Physiopathology of Obesity and Nutrition), 07122 Palma de Mallorca, Spain; lauragala3@gmail.com (L.G.-A.); mar.bibiloni@uib.es (M.d.M.B.); eargelich15@gmail.com (E.A.); eangullo@yahoo.es (E.A.-M.); cristina.bouzas@uib.es (C.B.); 2 Hospital de Manacor, IBSalut, 07500 Manacor, Spain; 3 Escola Graduada Primary Health Care Center, IBSalut, 07002 Palma de Mallorca, Spain

**Keywords:** metabolic syndrome, physical activity, functional fitness, older adults

## Abstract

Background: It has been pointed out that moderate to vigorous exercise improves metabolic syndrome (MetS) criteria; however, studies on functional fitness in subjects with MetS are scarce. Aim: This study aimed to assess functional fitness abilities in MetS and non-MetS subjects. Methods: Cross-sectional study. Participants living in the Balearic Islands (*n* = 477, 52% men, 55–80 years old) with MetS (n = 333) and without MetS (n = 144). Anthropometric, socioeconomic and lifestyle characteristics were measured, and blood samples were collected. Functional fitness tests included: one leg balance, standing and sitting handgrip, 30-s chair stand, arm curl, chair sit-and-reach, back scratch, 8-foot time up-and-go, 30-m walk, and 6-min walk tests. A Functional Fitness Score was created from tests that measured agility and dynamic balance, static balance, lower-and-upper body strength, lower-and-upper body flexibility, aerobic endurance, and speed. Results: All functional fitness tests were lower in MetS subjects, except for back scratch and standing handgrip test. After adjusting for possible confounders (sex, age, civil status, education level, leisure-time physical activity) MetS subjects were more likely to be below average for a sex and age specific cut-off value of one leg balance (Odds Ratio, OR: 2.37; 95% Confidence Interval, CI: 1.25–4.48), chair stand (OR: 2.30; 95% CI: 1.26–3.20), arm curl (OR: 3.43; 95% CI:1.90–6.26), back scratch (OR: 3.49; 95% CI: 2.31–5.91), 8-foot up-&-go (OR: 13.03; 95% CI: 6.66–25.55), 30-m walk (OR: 8.10; 95% CI: 4.33–15.57) and 6-min walk test (OR: 3.28; 95% CI: 1.76–6.52), whereas they were more likely to be above average for sitting handgrip test (OR:1.69; 95% CI:1.21–2.95). Functional Fitness Score was lower in MetS subjects (5.44 ± 2.40 vs. 7.04 ± 1.72, *p* < 0.001), independently of sex and age. Conclusion: MetS participants showed lower functional fitness abilities and lower Functional Fitness Score than non-MetS peers, independently of sex, age, body mass index and waist circumference, showing lower ability to perform everyday activities safely and independently.

## 1. Introduction

Metabolic syndrome (MetS) is a cluster of risk factors, including hypertension, dyslipidaemia, hyperglycaemia, and visceral fat [1,2] that raises the risk of developing cardiovascular disease (CVD) and type 2 diabetes mellitus (T2DM). MetS is responsible for 2.5-fold increased cardiovascular mortality, 2-fold increase in the risk of coronary heart disease and cerebro-vascular disease, 1.5-fold increase in the risk of all-cause mortality and 5-fold higher risk of developing diabetes [2,3].

The average prevalence of MetS in the United States is around 35% of all adults and 47% of those aged 60 years or older [4]. The average prevalence of MetS in Spain is around 31%, with Balearic Islands as one region with the highest prevalence (33.5%) [5]. These are alarming data, as 13% of the worldwide population is estimated to be 65 years or older by 2030, which means 8.3 billion people [6,7]. The global aging population is an important contributor to the increasing prevalence of MetS, as older adults are frequently affected by cardiovascular and metabolic risk factors that constitute the syndrome [7].

With advancing age, functional deterioration occurs even in the absence of disease [8]. Maintaining or improving functional fitness, defined as “having the physiological ability to perform everyday activities safely and independently without undue fatigue” [9] is an increasingly important goal with the aging of the world population. Functional fitness tests measure fitness parameters (balance, agility, speed, strength, flexibility, and aerobic endurance) using functional movement tasks [10]. There is evidence that aerobic training improved chair sit-and-reach and the 30-s chair stand performance and combined aerobic and resistance training ameliorated functional fitness (back scratch, chair sit-and-reach, 30-s chair stand, arm curl, 8-ft up-and-go, 6-min walk) in older adults [11]. In this way, regular practise of physical activity has demonstrated to reduce body weight and blood pressure, and upgraded lipid disorders, including raising high density lipoprotein cholesterol (HDL-c) and lowering triglycerides (TG) [12]. Exercise training improves body composition, cardiovascular and metabolic outcomes in people with MetS [13]. Physical activity should be considered as an essential part of lifestyle change in people with MetS, as the evidence shows that it ameliorates insulin resistance and the entire cluster of metabolic risk factors [14].

In industrialised countries where people are living longer lives, chronic health conditions are increasing, while few older adults achieve the level of exercise that accompanies health improvements [15]. The evidence showed that long-term, moderate to moderately vigorous intensity exercise training decreases MetS symptoms (raising high density lipoprotein-cholesterol and lowering triglycerides, and blood pressure and hypertension) [14]; thus, improving functional fitness abilities seems to be a high contributor to decrease the incidence of MetS and contributes to healthy aging. In this way, given the lack of studies measuring functional fitness in these subjects with MetS, the aim of the present study was to assess functional fitness abilities in MetS and non-MetS subjects.

## 2. Methods

### 2.1. Study Design, Sample, and Ethics

The cross-sectional study population comprised 477 community-dwelling adults of the Balearic Islands, men (52%) aged 55–80 years and women aged 60–80 years with no previously documented CVD (the sex-age range was chosen depending on the age that each gender is at high risk of suffering non-communicable diseases, the association of MetS with CVD, and the increasing prevalence of MetS with age [16]). Exclusion criteria included being institutionalized, suffering from a physical or mental illness which would limit fitness assessment or the ability to respond to questionnaires, chronic alcoholism or drug addiction, and inclusion in a clinical trial involving drug treatment over the past year. The study protocol and procedures were performed according to the ethical standards of the Declaration of Helsinki and were approved by the All participants provided written informed consent prior to participation.

### 2.2. Anthropometric and Blood Pressure Measurements

Anthropometric variables were measured by trained personnel to minimize the inter-observer coefficients of variation. Height was measured using a wall-mounted stadiometer, to the nearest millimetre, with the subject’s head in the Frankfurt Horizontal Plane position. Body weight was measured with high-quality electronic calibrated scales. Participants were weighed in bare feet and light clothes, subtracting 0.6 kg for their clothes. Body mass index (BMI) was calculated as weight in kilograms divided by the square of height in meters (kg/m^2^). Waist circumference (WC) was measured halfway between the last rib and the iliac crest by using an anthropometric tape [17]. Blood pressure was measured with a validated semi-automatic oscillometer (Omron HEM-705CP, Hoofddorp, The Netherlands) after 5 min of rest in-between measurements while the participant was in a seated position. All anthropometric variables were determined in duplicate, except for blood pressure which was measured in triplicate; the average score was used for analyses.

### 2.3. Blood Collection Analysis

Samples of blood were collected at 08:00 a.m. from antecubital vein after 8 h overnight fast. Biochemical analyses included fasting plasma glucose, and total cholesterol, HDL-cholesterol (HDL-c), and triglyceride (TG) concentrations measured in serum on the Abbott ARCHITECT c16000 employing commercial kits which included internal controls (Abbott Diagnostics, IL, USA).

### 2.4. Other Health Outcomes

Information regarding socioeconomic and lifestyle aspects (education level, civil status, and leisure-time physical activity) was collected. Educational level was ranked into primary school studies, secondary school studies and university graduate. Civil status was ranked into single, married, divorced and widow/er. Leisure-time physical activity (LTPA) was calculated as previously described [18], using the validated Spanish version of the Minnesota Leisure Time Physical Activity Questionnaire [19,20]. Alcohol intake was assessed by means of a semiquantitative 137-item food frequency questionnaire (FFQ) validated in Spain [21]. Smoking (>1 cigarette per day) was also registered.

### 2.5. Metabolic Syndrome Classification

Participants were classified as “with metabolic syndrome (MetS)” (n = 333) and “without MetS (non-MetS)” (n = 144) according to the updated harmonized definition of the International Diabetes Federation and the American Heart Association and National Heart, Lung and Blood Institute [3]. MetS is diagnosed if any 3 of 5 risk factors are present: high waist circumference (WC; ≥102 cm in men and ≥88 cm in women); elevated TG (≥150 mg/dL (1.7 mmol/L)) and/or drug treatment for elevated TG; reduced HDL-c (<40 mg/dL (1.0 mmol/L) in men and <50 mg/dL (1.3 mmol/L) in women) and/or drug treatment for reduced HDL-c; elevated blood pressure (systolic ≥130 and/or diastolic ≥85 mmHg) and/or antihypertensive drug treatment; elevated fasting glucose ≥100 mg/dL (5.6 mmol/L) and/or drug treatment for elevated glucose [3,22].

### 2.6. Functional Fitness Tests

The functional fitness tests administered in this study measure physical parameters using functional movement tasks [10]. Therefore, the 30-s chair stand test and the arm curl test (holding a hand weight of 2.5 kg for women and 4 kg for men) assess lower- and upper-body strength, respectively; the chair sit-&-reach and back scratch test measure lower- and upper-body flexibility, respectively; the 8-foot time up-&-go test (8-f TUG) measures motor agility and dynamic balance, the 6-min walk test assesses aerobic endurance [9,10,23], the 60-s one leg balance test [24] measures static balance, the standing and sitting handgrip test [25,26] assesses static strength of grip muscles and the 30-m walk test [27] measures speed. Handgrip test was measured with a handgrip dynamometer (Takei TKK 5401, Tokyo, Japan, range = 5–100 kg, precision = 0.1 kg).

All the above tests were performed on the same day and in the same order for each participant and supervised by trained personnel to minimize the inter-rate variability. A health-evaluation questionnaire was conducted prior to testing to ensure the safety of participants during exercise [23]. Instructions in every test were standardized to ensure that the same verbal information was given to all participants. All tests were performed twice, except for the 6-min walk test and the chair stand test, and the best score was retained.

Results of each functional fitness test associated with functional movement tasks were compared with the normal range of scores, considering age and sex [9,25,28]. A score above the normal range, was considered above average, and a score below the range was considered below average. Normal range was defined as the middle 50% of the population [9].

### 2.7. Functional Fitness Score

A Functional Fitness Score was built from functional movement tasks, measuring physical parameters (agility and dynamic balance, static balance, lower- and upper-body strength, static strength of grip muscles, lower- and upper-body flexibility, aerobic endurance, and speed) to assess a wide range of ability levels that are essential for optimal health and to perform normal everyday activities safely and independently without undue fatigue [9,10]. Functional fitness score ranged from 0 to 9 and was based on a sex and age specific cut-off value of functional fitness tests: chair stand, arm curl, 6-min walk, chair sit-and-reach, back scratch, 8-f TUG test [9], the 60-s one leg balance test [28], standing and sitting handgrip test [25] and 30-m walk test [28]. Scores below the normal range were assigned a value of 0 points, and those above the normal range +1 point, indicating that for that functional movement task, functional fitness level of the participant was appropriate or above average for their sex and age.

### 2.8. Statistics

Analyses were performed with the Statistical Package for the Social Sciences version 25.0 (IBM SPSS Statistics for Windows, Chicago, IL, USA). All tests were stratified by MetS status and sex. Normality of data was assessed by the Kolmogorov-Smirnov test and visual inspection of histograms and normal probability plots. Categorical variables were presented as frequencies and/or proportions. Significant differences in prevalence were calculated by chi-squared test. Continuous variables were presented as mean and standard deviation (SD) or median and interquartile range (IQR) and significant differences were tested by unpaired Students’ *t*-test or Mann-Whitney test. Equality of variances was assessed with Levene’s test. Logistic regression analysis with the estimation of the corresponding odds ratio (OR) and the 95% Confidence Interval (95% CI) were calculated (Table 3) to examine the association between functional fitness tests (dependent variables) and MetS subjects above and below average for a sex and age specific cut-off value compared with non-MetS ones (independent variables). Univariate analysis was first carried out (crude OR). Secondly (OR adjusted 1), results were adjusted for sex, age (continuous variable). Thirdly (OR adjusted 2), results were adjusted for sex, age (continuous variable), civil status, education level, smoking, LTPA (continuous variable, expressed in MET*min/day), and BMI (continuous variable) to control for potential confounders. Results were considered statistically significant if *p*-value (2 tailed) < 0.05.

## 3. Results

Characteristics of participants with and without MetS are shown in Table 1. Sample was 144 participants without MetS (43.8% men) and 333 participants with MetS (55% men). Significant differences were found in sex, weight, BMI, WC, prevalence of obesity, LTPA, current smoking, diabetes, and hypertension between groups. Criteria of MetS were mostly shown by MetS participants. No differences were observed in age, height, civil status, educational level, alcohol consumption, and HDL-c.

Functional fitness tests. Table 2 shows functional fitness tests results in MetS and non-MetS subjects. There were significant differences between MetS and non-MetS subjects in all variables except for chair sit-&-reach test and standing handgrip. No differences were observed in sitting handgrip in women. Individuals with MetS had lower scores for the one leg balance, chair stand, arm curl, back scratch, 8-f TUG, 30-m walk, and 6-min walk test, except for sitting handgrip in men.

Table 3 shows the comparison of MetS subjects above and below average for a sex and age specific cut-off value of functional fitness tests with non-MetS subjects as reference value. After adjusting for possible confounders, MetS participants were significantly more likely to be below average for one leg balance (2.37 odds), chair stand (2.30 odds), arm curl (3.43 odds), back scratch (3.49 odds), 8-foot up-&-go (13.03 odds), 30-m walk (8.10 odds) and 6-min walk test (3.28 odds), whereas they were significantly more likely to be above average for sitting handgrip (1.69 odds) compared with non-MetS subjects. No significant differences were found in standing handgrip and chair sit-&-reach test.

Functional Fitness Score. Functional Fitness Score considering a sex and age specific cut-off value for each functional fitness test is shown in Table 4 Functional Fitness Score was significantly lower in MetS (5.44 ± 2.40) than in non-MetS group (7.04 ± 1.72, *p* ≤ 0.001), independently of sex, age, BMI, and WC. However, when subjects were normal weight (BMI < 25 kg/m^2^), and WC was higher than 102 cm (men) and 88 cm (women), there were no differences between MetS and non-MetS participants.

## 4. Discussion

The most relevant observation of the current study was that MetS participants showed lower Functional Fitness Score than non-MetS subjects independently of sex, age, BMI, and WC. MetS participants showed lower performance in upper-body and lower-body strength, aerobic endurance, upper-body flexibility, agility and dynamic balance, speed, and static balance, independently of sex and age, resulting in a lower common mobility and higher risk of disability [10]. MetS subjects were more likely to be below average for one leg balance, chair stand, arm curl, back scratch, 8-foot up-&-go, 30-m walk, and 6-min walk test. These results are related with lower capacity to perform functional movements, such as walking, stair climbing and standing up, required for everyday living, and compromising, in this way, their independence [9,10,28].

Functional Fitness Score. A Functional Fitness Score is proposed as a new tool to evaluate functional fitness, built on fitness parameters that support functional mobility in these subjects. Functional Fitness Score in non-MetS group was higher than in the MetS group for age, sex, BMI, and WC, which could be related with lower ability to perform everyday activities safely and independently in MetS group [9,10], which agrees with the lack of differences between MetS and non-MetS subjects at normal weight and non-risk. There is evidence that interventions on MetS patients including exercise training, improve MetS risk factors [29,30]; it demonstrates that regular exercise training may also ameliorate functional fitness in this cohort [11]. Then, improved functional fitness will enhance MetS risk factors, and then it will upgrade health.

Agility, balance, and speed. Balance and agility deficits result in multiple complications and are associated with the increased incidence of falls seen in the older adult population that are related with significant morbidity and mortality [31,32]. Static balance and walking speed are considered important to evaluate lower body performance [28]. Duration of standing position on one leg balance test diminishes with age [28] and it was significantly lower in MetS group. Evidence shows that the one-leg balance test appears to be a significant predictor of fall-related injuries [32] and it is related to declines in activity of daily living and other morbidity [33,34], indicating that MetS subjects were more likely to have difficulty keeping balance that may eventually lead to a higher probability of falls [33,34]. A limitation of this test was the limit time of 60 s, because there are people who could have continued more than this limit time, so the mean time does not reflect the reality. This should be considered in future studies. In fact, there are other studies that set the limit time in 120 s [35,36].

Time to complete the 30-m walk test was higher in MetS subjects indicating a lower walking speed in MetS participants, which could have several consequences. Evidence has been provided that slow walking speed was a strong predictor of increased risk of ischemic stroke among postmenopausal women independent of other stroke established risk factors [37]. Furthermore, evidence shows a 2-fold increased risk of mortality for individuals with slower walking speed [38], with gait speed being a useful clinical tool in the prediction of dementia [39,40].

8-f TUG test is a good measure of combined physiological attributes (power, speed, agility, and dynamic balance) [10]. Time to complete 8-f TUG test was higher in MetS subjects, as they were 13.03 times more likely to be below average than non-MetS peers, showing lower common mobility and gait manoeuvres, required in independent living activities. Comparing the results of our MetS sample with previous reported values in ≥65-year-old-adults [40], our participants showed lower agility and dynamic balance performance.

Aerobic endurance. The 6-min walk test provides information regarding aerobic endurance in aging population [10,28], which is essential to maintain a good cardiometabolic health [41,42]. Distance completed in the 6-min walk test was shorter in MetS subjects, showing a lower aerobic endurance performance. It compromises the aerobic ability to perform daily activities as walking, shopping, etc. [10,28]. Comparing the results of our MetS sample with previous reported values of independent non-institutionalized >65-year-old adults [28] and physically active ≥65-year-old people [43], our MetS participants showed lower aerobic endurance. Nevertheless, comparing with other reported results in sedentary women (>60 years old), our participants showed higher aerobic endurance [44].

Lower- and upper-body flexibility. Reduced range of movement in the shoulder may result in pain and postural instability [10] and may cause significant disability in around 30% of healthy adult population older than 60 years [10,45]. Upper-body flexibility in MetS subjects was lower than non-MetS peers, which is associated with the development of musculoskeletal impairments and the progression of disabilities in the elderly [44]. Although upper-body flexibility declines with age, the evidence shows that older adults still have the capacity to improve flexibility with exercise training programs [46].

Body strength. Lower body muscular integrity is important to maintain functional mobility and preventing or delaying the onset of disability [10]. The chair stand test provides information on declines in mobility and a measure to identify frail individuals [47], being an effective screening tool for sarcopenia [48]. Lower number of repetitions in the chair stand test were observed in MetS subjects, showing reduced lower-body performance in this group, compromising functional capacity and mobility [44]. Furthermore, compared to previous published data of Mexican Americans aged 65 years or more [38] and sedentary women with and without MetS (>60 years old) [44], MetS subjects showed less repetitions in the chair stand test.

Handgrip strength is correlated with functional fitness and frailty among older population [49]. Higher grip strength score was obtained in the sitting handgrip test in MetS males compared to non-MetS males, which is correlated with improved functional ability. Nonetheless, no significant differences were found in standing handgrip, and previous data on healthy 310 male and 328 female adults aged 20–94 years showed higher grip strength than MetS subjects for both genders and all ages [50]. On the contrary, compared with Asian subjects aged ≥60 years [49], MetS subjects showed higher grip strength scores for men and lower grip strength scores for women. Besides, compared with a previous study of physically active adults aged 65 years or older, our participants showed higher grip strength [43]. However, due to the lack of significance in standing handgrip, more studies are needed.

The arm-curl test is a common measurement test of upper extremities’ strength in older age and has been correlated with general muscular endurance [51]. Muscular endurance is the ability of muscles to work for long periods without undue fatigue, preventing unwanted fatigue in daily routines [10]. MetS showed less repetitions in the arm curl test, resulting in a reduced upper-body strength among MetS participants, compromising the ability to execute normal everyday activities such as household chores, carrying groceries and picking up grandchildren [10]. Comparing our results with previous published data of Mexican Americans aged 65 years or more [38], sedentary women with and without MetS (>60 years old) [44] and ≥65 years old [40], MetS group showed lower–upper-body strength [40,44], independently of age and sex [38].

It has been pointed out that smoking, alcohol, comorbidities such as diabetes and hypertension, and BMI can substantially affect physical condition and fitness, mainly on the movement of lower body [52,53,54,55,56]. BMI also showed positive association with grip strength, vertical jump, and push-ups [57]. Favourable levels of relative time spent in lifestyle movement behaviours were, in general, associated with decreased BMI [58]. However, the current data were not affected after adjustments by these outcomes, showing an association between lower fitness parameters and MetS severity. These results agree previous studies pointing out that MetS components are positively influenced by physical activity, and then exercise therapy is an effective intervention to both prevent and mitigate the impact of MetS [12]. Therefore, physical activity should be part of treatment strategies for MetS. The challenge for health care professionals now will be how to motivate individuals to adhere and participate in programmes of exercise to treat the MetS [59].

## 5. Strengths and Limitations

To our knowledge, the main strength of the current study was that this is the first study assessing functional fitness with this complete test battery in older adults with and without MetS. Moreover, the Functional Fitness Score provides a useful tool to evaluate functional fitness in these subjects. However, this study has several limitations. First, the cross-sectional study limits the ability to elucidate a cause-effect relationship between worse functional fitness and the presence of MetS. Second, the one leg balance test limits the time to 60 s, so the mean time of this test does not reflect the reality, because there are people who could have continued more than this time. It has been previously concluded that durations of less than 120 s appear to be insufficient to identify balance limitations in many adults less than 80 years of age [33]. Third, the sample size is unequal between groups (MetS and non-MetS subjects). Fourth, as functional fitness was assessed in older population, generalizability to other populations with different age ranges may be limited.

## 6. Conclusions

MetS participants showed lower functional fitness abilities and lower Functional Fitness Score than non-MetS peers, independently of sex, age, BMI, and WC, showing lower ability to perform everyday activities safely and independently. Accordingly, subjects with MetS would need care on dietary but also on daily physical activity to maintain their functional fitness, as an important contributor to their health status.

## Figures and Tables

**Table 1:** Characteristics in older adults between MetS and non-MetS participants.

	MetS (n = 333)	Non-MetS (n = 144)	*p-*Value
**Men (%)**	55.0	43.8	0.028
**Age, y**	64.9 ± 5.4	65.5 ± 5.6	0.342
**Weight, kg**	86.2 ± 14.0	69.1 ± 12.5	<0.001
**Height, cm**	163.1 ± 9.3	162.4 ± 8.8	0.482
**BMI, kg/m^2^**	32.4 ± 3.87	26.1 ± 3.3	<0.001
**Waist circumference, cm**	108.8 ± 11.4	85.4 ± 10.9	<0.001
**Prevalence of obesity (%)**	94.8	5.2	<0.001
**Civil status (%)**			0.137
**Single**	5.0	3.5	
**Married**	77.7	70.1	
**Divorced**	6.8	9.0	
**Widow/er**	10.5	17.4	
**Education level (%)**			0.179
**Primary**	47.4	47.2	
**Secondary**	32.2	34.7	
**University graduate**	20.4	18.1	
**Total LTPA (MET·min/day)**	518.5 ± 411.3	883.6 ± 1200.8	<0.001
**Alcohol intake (g/day)**	199.3 ± 174.11	160.9 ± 157.1	0.088
**Current smoking (%)**	13.9	6.3	0.017
**Diabetes (%)**	73.8	26.2	<0.001
**Hypertension (%)**	91.8	72.5	<0.001
**Meeting MetS criteria (or with treatment)**			
**WC ≥ 102 cm (men) ≥ 88 cm (women) (%)**	97.4	27.1	<0.001
**TG ≥ 150 mg/dL (%)**	54.4	15.5	<0.001
**HDL-c < 40 mg/dL (men) <50 mg/dL (women) (%)**	47.4	39.1	0.071
**Blood pressure (systolic ≥130; diastolic ≥85 mmHg) (%)**	91.5	79.2	<0.001
**Fasting glucose ≥100 mg/dL (%)**	78.5	32.9	<0.001

Abbreviations: BMI: body mass index; HDL-c: high density lipoprotein-cholesterol; LTPA: leisure-time physical activity; MET: Metabolic Equivalent; MetS: Metabolic Syndrome; TG: triglycerides; WC: waist circumference. Difference in means (±SD) were tested by unpaired Students’ *t* test and differences in percentages were tested by chi-squared test.

**Table 2:** Functional fitness tests in older adults (55–80 years) between MetS and non-MetS participants.

		MetS (n = 333)		Non-MetS (n = 144)		*p*-Value
		Mean ± SD	Median (IQR)	Mean ± SD	Median (IQR)	
**One leg balance test (s)**	Men	39.5 ± 20.7	44.0 (38.6)	46.0 ± 19.4	60.0 (34.9)	0.022
	Women	29.8 ± 21.2	22.8 (46.6)	41.3 ± 21.3	60.0 (39.7)	<0.001
	Total	35.3 ± 21.4	34.4 (45.9)	43.4 ± 20.5	60.0 (37.0)	<0.001
**Standing handgrip (Kg)**	Men	38.5 ± 8.0	38.1 (10.7)	37.3 ± 6.3	36.3 (10.5)	0.196
	Women	20.9 ± 5.5	20.4 (7.9)	20.7 ± 4.4	20.1 (6.2)	0.729
	Total	30.7 ± 11.2	30.0 (17.8)	27.9 ± 9.8	26.10 (15.1)	0.019
**Sitting handgrip (Kg)**	Men	38.3 ± 8.0	38.5 (10.6)	36.4 ± 5.7	36.2 (9.7)	0.045
	Women	20.6 ± 5.5	20.4 (7.1)	20.1 ± 4.4	20.4 (5.7)	0.634
	Total	30.5 ± 11.2	29.2 (17.8)	27.3 ± 9.5	25.0 (14.9)	0.006
**Chair stand test (reps)**	Men	13.5 ± 3.7	13.0 (4.0)	15.3 ± 3.2	15.0 (5.0)	<0.001
	Women	12.1 ± 3.1	12.0 (4.0)	13.8 ± 3.3	14.0 (4.0)	<0.001
	Total	12.9 ± 3.5	13.0 (4.0)	14.4 ± 3.3	14.0 (5.0)	<0.001
**Armcurl test (reps)**	Men	16.2 ± 4.4	16.0 (6.0)	19.7 ± 4.9	19.0 (7.0)	<0.001
	Women	15.2 ± 4.4	15.0 (6.0)	17.3 ± 3.5	18.0 (5.5)	<0.001
	Total	15.7 ± 4.4	15.0 (5.9)	18.3 ± 4.3	18.0 (6.0)	<0.001
**Chair Sit-&-Reach test (cm)**	Men	−3.3 ± 9.3	−1.0 (11.8)	−0.6 ± 10.0	0.0 (12.5)	0.070
	Women	−2.3 ± 8.2	0.0 (10.0)	−1.3 ± 9.2	0.0 (11.5)	0.608
	Total	−2.9 ± 8.8	0.0 (10.0)	−1.0 ± 9.6	0.0 (12.0)	0.085
**Back Scratch test (cm)**	Men	−13.6 ± 11.1	−14.0 (15.9)	−5.4 ± 9.4	−3.0 (13.5)	<0.001
	Women	−9.4 ± 9.3	−8.00 (13.0)	−0.7 ± 7.0	1.0 (9.0)	<0.001
	Total	−11.7 ± 10.5	−10.5 (15.0)	−2.1 ± 8.4	0.0 (12.0)	<0.001
**8-Ft Up-&-Go test (s)**	Men	6.0 ± 1.5	5.8 (1.7)	4.8 ± 0.8	4.8 (0.9)	<0.001
	Women	7.1 ± 2.2	6.7 (2.1)	5.4 ± 0.8	5.3 (1.4)	<0.001
	Total	6.5 ± 1.9	6.2 (1.9)	5.2 ± 0.9	5.0 (1.2)	<0.001
**30-m walk test (s)**	Men	17.2 ± 4.1	16.9 (5.0)	14.4 ± 2.5	14.3 (3.4)	<0.001
	Women	21.0 ± 5.1	20.4 (6.8)	16.8 ± 2.1	16.7 (3.6)	<0.001
	Total	18.8 ± 4.9	18.3 (6.3)	15.8 ± 2.6	15.6 (3.5)	<0.001
**6-min walk test (m)**	Men	563.9 ± 82.8	565.8 (101.2)	627.1 ± 92.6	625.6 (119.6)	<0.001
	Women	487.0 ± 76.2	492.2 (82.8)	534.8 ± 62.5	533.6 (73.6)	<0.001
	Total	532.8 ± 88.4	529.0 (113.9)	575.2 ± 94.1	563.5 (115.0)	<0.001

Abbreviations: cm: centimetre; MetS: Metabolic Syndrome; reps: repetitions; s: seconds; m: metre; min: minutes. Difference in means between MetS and non-MetS group were tested by unpaired Student’s *t* test. Negative numbers in the chair sit-&-reach test and the back scratch test means no reaching the toes or the extended middle fingers, respectively.

**Table 3:** Logistic regression models for age and sex specific cut-off values * of functional fitness tests (dependent variables) in MetS and non-MetS participants (independent variables).

		Non-MetS (n = 144)			Mets (n = 333)				
		Above Average	Below Average			Above Average		Below Average	
		n	n	OR (95% CI)	n	OR (95% CI)	n	OR (95% CI)	*p-*value
**One leg balance test (s)**	Crude OR	127	*16*	1.00 (ref.)	231	0.38 (0.21–0.69)	76	2.61 (1.46–4.67)	0.001
	OR adjusted 1			1.00 (ref.)		0.37 (0.21–0.66)		2.71 (1.51–4.87)	0.001
	OR adjusted 2			1.00 (ref.)		0.39 (0.19–0.68)		2.37 (1.25–4.48)	0.008
**Standing handgrip (Kg)**	Crude OR	64	*80*	1.00 (ref.)	154	1.20 (0.81–1.79)	160	0.83 (0.56–1.24)	0.360
	OR adjusted 1			1.00 (ref.)		1.19 (0.80–1.77)		0.84 (0.57–1.26)	0.404
	OR adjusted 2			1.00 (ref.)		1.39 (0.85–2.19)		0.75 (0.48–1.20)	0.223
**Sitting handgrip (Kg)**	Crude OR	59	*85*	1.00 (ref.)	157	1.44 (0.97–2.15)	157	0.69 (0.47–1.04)	0.073
	OR adjusted 1			1.00 (ref.)		1.41 (0.94–2.11)		0.71 (0.47–1.06)	0.095
	OR adjusted 2			1.00 (ref.)		1.69 (1.21–2.95)		0.63 (0.39–0.86)	0.037
**Chair stand test (reps)**	Crude OR	119	*24*	1.00 (ref.)	195	0.32 (0.20–0.52)	123	3.13 (1.91–5.12)	<0.001
	OR adjusted 1			1.00 (ref.)		0.33 (0.20–0.54)		3.03 (1.85–4.98)	<0.001
	OR adjusted 2			1.00 (ref.)		0.43 (0.26–0.72)		2.30 (1.26–3.20)	0.003
**Arm curl test (reps)**	Crude OR	128	*16*	1.00 (ref.)	208	0.24 (0.14–0.43)	108	4.15 (2.35–7.34)	<0.001
	OR adjusted 1			1.00 (ref.)		0.25 (0.14–0.45)		3.95 (2.22–7.03)	<0.001
	OR adjusted 2			1.00 (ref.)		0.28 (1.16–0.55)		3.43 (1.90–6.26)	<0.001
**Chair Sit-&-Reach test (cm)**	Crude OR	83	*61*	1.00 (ref.)	181	0.98 (0.66–1.46)	136	1.02 (0.69–1.52)	0.913
	OR adjusted 1			1.00 (ref.)		0.96 (0.65–1.44)		1.04 (0.69–1.55)	0.857
	OR adjusted 2			1.00 (ref.)		1.15 (0.66–1.78)		0.89 (0.63–1.341)	0.489
**Back Scratch test (cm)**	Crude OR	93	*51*	1.00 (ref.)	94	0.24 (0.16–0.36)	217	1.21 (2.77–6.40)	<0.001
	OR adjusted 1			1.00 (ref.)		0.24 (0.16–0.37)		4.17 (2.74–6.36)	<0.001
	OR adjusted 2			1.00 (ref.)		0.28 (0.18–0.46)		3.49 (2.31–5.91)	<0.001
**8-foot Up-&-Go test (s)**	Crude OR	132	*12*	1.00 (ref.)	142	0.07 (0.04–0.14)	179	13.87 (7.38–26.05)	<0.001
	OR adjusted 1			1.00 (ref.)		0.07 (0.04–0.13)		14.70 (7.77–27.82)	<0.001
	OR adjusted 2			1.00 (ref.)		0.09 (0.06–0.18)		13.03 (6.66–25.55)	<0.001
**30-m walk test (s)**	Crude OR	129	*15*	1.00 (ref.)	110	0.11 (0.06–0.20)	114	8.91 (4.91–16.17)	<0.001
	OR adjusted 1			1.00 (ref.)		0.11 (0.06–0.20)		9.23 (5.04–16.91)	<0.001
	OR adjusted 2			1.00 (ref.)		0.13 (0.07–0.25)		8.10 (4.33–15.57)	<0.001
**6-min walk test (m)**	Crude OR	126	*18*	1.00 (ref.)	104	0.29 (0.16–0.52)	52	3.50 (1.93–6.35)	<0.001
	OR adjusted 1			1.00 (ref.)		0.29 (0.16–0.54)		3.41 (1.87–6.24)	<0.001
	OR adjusted 2			1.00 (ref.)		0.32 (0.18–0.60)		3.28 (1.76–6.52)	<0.001

Abbreviations: OR. Odds Ratio. Logistic regression analysis was used to examine the association between functional fitness tests and MetS subjects above and below average for a sex and age specific cut-off value compared with non-MetS ones. OR adjusted 1: Odds Ratio adjusted by sex and age. OR adjusted 2: Odds Ratio adjusted by sex, age, civil status, education level, smoking, total leisure-time physical activity, and BMI. * According to Rikli and Jones (31), Pedrero-Chamizo (32) and Dodds (29).

**Table 4:** Functional Fitness Score considering a sex and age specific cut-off value for functional fitness.

	Mets (n = 333)	Non-MetS (n = 144)	
	Mean ± SD	Mean ± SD	*p-*value
**Total**	5.44 ± 2.40	7.04 ± 1.72	<0.001
**Men**	5.40 ± 2.28	6.90 ± 1.81	<0.001
**Women**	5.49 ± 2.59	7.14 ± 1.66	<0.001
**Age (years)**			
**55–64**	5.55 ± 2.27	7.02 ± 1.82	<0.001
**65–69**	5.45 ± 2.53	7.15 ± 1.44	0.001
**≥70**	5.11 ± 2.56	6.91 ± 1.91	0.002
**BMI (kg/m^2^)**			
**<25**	4.20 ± 2.05	5.06 ± 1.20	0.160
**25–<30**	4.18 ± 1.32	4.65 ± 1.33	0.050
**≥30**	3.02 ± 1.77	4.23 ± 1.23	0.019
**WC (cm)**			
**<102 (men) or <88 (women)**	3.65 ± 1.72	4.80 ± 1.30	<0.001
**≥102 (men) ≥88 (women)**	3.18 ± 1.69	3.86 ± 1.10	0.303

BMI: body mass index; MetS: Metabolic Syndrome; WC: waist circumference. Differences in Functional fitness score between MetS and non-MetS participants were tested by Mann-Whitney test.

## Data Availability

There are restrictions on the availability of data for this trial, due to the signed consent agreements around data sharing, which only allow access to external researchers for studies following the project purposes. Requestors wishing to access the trial data used in this study can make a request to pep.tur@uib.es.
